# FANCI is Associated with Poor Prognosis and Immune Infiltration in Liver Hepatocellular Carcinoma

**DOI:** 10.7150/ijms.83760

**Published:** 2023-05-12

**Authors:** Yibo Hou, Jianing Li, Albert Yu, Kexin Deng, Jiawei Chen, Zixian Wang, Laiqiang Huang, Shaohua Ma, Xiaoyong Dai

**Affiliations:** 1 Institute of Biopharmaceutical and Health Engineering, Shenzhen Key Laboratory of Gene and Antibody Therapy, State Key Laboratory of Chemical Oncogenomics, Shenzhen International Graduate School, Tsinghua University, Shenzhen, Guangdong 518055, China.; 2 Guangdong Key Laboratory of Chiral Molecule and Drug Discovery, School of Pharmaceutical Sciences, Sun Yat-sen University, Guangzhou, Guangdong, 510006, China.

**Keywords:** Liver Hepatocellular carcinoma, FANCI, prognosis, tumor immune infiltration, chemoresistance

## Abstract

**Objective:** This study aimed to validate FANCI as a potential marker for both prognosis and therapy in liver hepatocellular carcinoma.

**Method:** FANCI expression data were acquired from GEPIA, HPA, TCGA, and GEO databases. The impact of clinicopathological features was analyzed by UALCAN. The prognosis of Liver Hepatocellular Carcinoma (LIHC) patients with highly expressed FANCI was constructed utilizing Kaplan-Meier Plotter. GEO2R was employed to identify differentially expressed genes (DEGs). Metascape was used to analyze functional pathways correlations. Protein-Protein interaction (PPI) networks were generated by Cytoscape. Furthermore, molecular complex detection (MCODE) was utilized to recognize Hub genes, which were selected to establish a prognostic model. Lastly, the relationship between FANCI and immune cell infiltration in LIHC was examined.

**Results:** Compared to adjacent tissues, FANCI expression levels were significantly higher in LIHC tissues and were positively correlated to the cancer grade, stage, and prior hepatitis B virus (HBV) infection. High expression of FANCI was found to be associated with poor prognosis in LIHC (HR=1.89, p<0.001). DEGs that were positively correlated with FANCI were involved in various processes, including the cell cycle, VEGF pathway, immune system processes, and biogenesis of ribonucleoproteins. MCM10, TPX2, PRC1, and KIF11 were identified as key genes closely related to FANCI and poor prognosis. A reliable five-variable prognostic model was constructed with strong predictive capability. Lastly, a positive correlation was observed between FANCI expression and tumor-infiltration levels of CD8+ T cells, B cells, regulatory T (Tregs), CD4+ T helper 2 (Th2), and macrophage M2 cells.

**Conclusion:** FANCI may hold promise as a potential biomarker for predicting prognostic outcomes, and a valuable therapeutic target for LIHC patients, with a focus on anti-proliferation, anti-chemoresistance, and combination with immunotherapy.

## Introduction

Liver cancer is a major public health concern, ranking sixth in incidence and third in mortality worldwide. In 2022, an estimated 41,260 new cases of liver cancer were diagnosed, representing 2.2% of all new cancer cases. Additionally, 30,520 patients would die from liver cancer, contributing 5.0% of all cancer in the United States 1. The incidence rate has been rapidly rising by 2% annually as of 2014, especially in women. The same trend has been observed in the mortality rate, which has risen by an average of 1.3% per year from 2010 to 2019. However, the 5-year relative survival rate has improved from 3% to 20.8% from 1980 to 2013. Notably, the early detection of liver cancer is key to achieving better long-term survival, with a survival rate of 35.3% in localized liver cancer 2. Liver cancer can be divided into hepatocellular carcinoma (HCC), cholangiocarcinoma, and mixed type. Among them, HCC is the most common malignancy of liver cancer. The treatment options are variable based on the different stages. Targeted therapy and immunotherapy have been developed for the treatment of advanced and recurrent liver cancer. Sorafenib has been approved by the Food and Drug Administration (FDA) as a first-line therapy. However, the results of studies investigating its use alone or in combination with other agents have been controversial 3. In addition to sorafenib, immunotherapy has also been explored as a treatment option for liver cancer. Pembrolizumab, an immune checkpoint inhibitor against PD-1, was approved by the FDA as a second-line therapy to treat hepatocellular carcinoma, with a reported 17% response rate 4. Nivolumab, another anti-PD-1 immune checkpoint inhibitor (ICI), presented a 14.3% overall response rate 5. Nevertheless, the response rates for all therapies remain relatively low, largely due to tumor heterogeneity of liver cancer. Thus, it is urgent to discover and elucidate the unique biomarkers related to tumor grouping, metastasis, prognosis, and treatment selection for liver cancer.

Fanconi anemia (FA) is a rare inherited disease that causes genomic instability, which significantly increases the predisposition to cancer. The mechanism of progression for FA is mediated by the FA family of 22 genes, including FANCA, FANCM, FANCU, FANCD2, FANCI, *etc.* 6, 7. Mutations of these genes can impair the repair of DNA interstrand cross-links (ICLs), which further leads to the development of FA. In the normal state, certain kinases such as ataxia telangiectasia and Rad3-related kinase (ATR) and checkpoint kinase 1 (CHK1) can receive signals relating to DNA damage and trigger the FA DNA damage response pathway by phosphorylating FANCE, FANCM, and FANCG. Moreover, the FA core complex, consisting of five other FA proteins, is recruited, which can monoubiquitinate the downstream FANCD2-FANCI complex. Subsequently, ATR, CHK1, and ATM phosphorylate the heterodimer, and the conformational change of the phosphorylated FANCI promotes the disassociation of the heterodimer 8. The ubiquitinated FANCI and FANCD2 can work independently or coordinately to repair distinct DNA damage to restart the cell cycle 9. The effect of the FA pathway, especially the FANCD2-FANCI heterodimer, is twofold in cancer progression. Initially, FA acts as a guardian to maintain the integrity of the genome, thereby preventing carcinogenesis. However, the overexpression of the FANCI or FANCD2 can lead to increased repair of DNA damage. Which may contribute to cancer cells' survival, and promote tumor cell resistance against radiotherapy and chemotherapy 10. Besides, the functions of the FANCI protein have been investigated in various cancers, and FANCI has also been proven to be regarded as a prognostic biomarker, given its high expression is associated with poor survival rate and therapeutic outcome in lung adenocarcinoma, cervical cancer, and ovarian cancer 11-13. However, there is currently a lack of studies on the relationship between the expression of FANCI and liver cancer. At the same time, we found high expression of FANCI is also associated with poor clinical outcomes. Thus, we aim to investigate the mechanism by which FANCI regulates the development and progression of LIHC.

To elucidate the impacts of abnormal expression of FANCI on the progression, development, metastasis, and prognosis of liver cancer, a comprehensive bioinformatics analysis was conducted. Initially, the mRNA and protein expression levels of FANCI in LIHC were investigated, and the prognosis situation of the patient was analyzed. Furtherly, the differentially expressed genes from GEO and TCGA databases were investigated, and their functions were explored via gene enrichment analysis. Key genes associated with FANCI were found, and their prognostic value and risk score were calculated. Eventually, the relationships between FNACI and immune infiltration in LIHC were evaluated.

## Material and Method

### GEPIA Database

The GEPIA web server (http://gepia.cancer-pku.cn) was utilized to obtain the gene expression profile of FANCI among the majority of the cancer types by analyzing 9736 tumors and 8587 normal RNA sequencing samples, which were collected from the TCGA and GTEx programs. The expression pattern of FANCI in LIHC was then determined by comparing it to normal samples from TCGA and GTEx. The overall survival (OS) and disease-free survival (DFS) curves based on the expression of FANCI in LIHC were constructed, and a Cox PH Model was used to calculate the hazards ratio (HR) with a 95% confidence interval.

### Human Protein Atlas (HPA) Database

The various expression profiles of FANCI in different cell types and tissues, both normal and tumor, were acquired from the HPA website (https://www.proteinatlas.org/), which maps protein and gene expression patterns in cells, tissues, and organs via omics tools. Additionally, the protein expression between the LIHC and normal tissue was determined using immunohistochemistry from the HPA database.

### UALCAN Database

The UALCAN database (http://ualcan.path.uab.edu) contains the gene expression data of 31 tumors gathered from the TCGA database. The website was used to explore the various FANCI expression profiles in LIHC based on clinicopathological characteristics such as the stage, grade, and histological subtype.

### GEO Database

The gene expression data regarding LIHC were downloaded from the GEO website, a public repository containing various forms of genetic information. GSE14520 and GSE45267 mRNA microarrays were extensively investigated, with GSE14520 containing 220 normal liver tissue samples and 225 liver tumor tissue samples and GSE45267 containing 41 normal liver tissue samples and 46 liver tumor tissue samples. The GEO2R database (https://www.ncbi.nlm.nih.gov/geo/geo2r/) was used to obtain the expression of FANCI in the normal and cancer samples. The results were then extracted, and box plot graphs were constructed using ggplot2 [version: 3.3.3] in R software. In addition, GEO data were analyzed to discover the DEGs in the GSE14520 microarray with adjusted P-value < 0.05, |log2FC |> 1, with the adjusted P-value efficiently correcting for false-positives. Eventually, a volcano plot was constructed by using imageGP (http://www.ehbio.com/ImageGP/).

The same threshold was utilized to determine the DEGs by analyzing the LIHC data from the TCGA database, which was completed with the help of the Limma package (version: 3.40.2) of R software. This study contained 371 LIHC samples and 50 tumor-adjacent tissue samples. The overlap of the upregulated and downregulated DEGs from the GEO and TCGA datasets was quantified using ggplot2, and a Venn diagram was used to depict the results.

### Prognostic Analysis

The prognostic data of FANCI for 31 types of cancer found in the TCGA was analyzed by the univariate Cox regression method and visualized using R software forest plots. Plots contained information about P-value, HR, and 95% Confidence interval (CI). TCGA data for LIHC was utilized to generate Kaplan-Meier curves indicating the OS, DFS (PFI), and disease-specific survival (DSS). 424 RNA-seq data from the LIHC patients were divided into high expression and low expression groups, then analyzed by survival (version: 3.2-10) and visualized by survminer package (version: 0.4.9) for R. The “surv_cutpoint” function was applied in the survminer to separate patients into two groups, obtaining the optimal threshold value with the lowest p-value. Furthermore, the TCGA-LIHC data was separated into HBV positive and HBV negative groups based on mRNA expression, and the OS curves were plotted by ggplot2.

### ICGC Dataset

The ICGC dataset was utilized to supplement the TCGA and GEO data. 240 primary liver cancer RNA-sequence data and 202 normal liver RNA-sequence data were studied using ggplot2 to assess the differential expression of FANCI. In addition, the KM curve was plotted to indicate the difference in survival rate between the high expression and low expression groups, which was achieved with the help of the survival and survminer R package.

### Gene enrichment Analysis

Gene enrichment analysis was conducted by Metascape (https://metascape.org/gp/index.html#/main/step1). Firstly, the overlapping upregulated and downregulated DEGs were analyzed to generate analysis reports, which included Gene Ontology (GO), Kyoto Encyclopedia of Genes and Genomes (KEGG) pathway, Reactome gene sets, and more*.*

Meanwhile, two gene lists were utilized to construct a PPI network facilitated by the string database (version: 11.5) (https://string-db.org/), with high confidence (0.700) and active interaction obtained from textmining, experiments, databases, and co-expression. Subsequently, PPI networks were generated and visualized by Cytoscape (Version: 3.9.0) with the default style. In addition, the MCODE tool, a Cytoscape plugin, was employed to search Hub genes within the PPI networks. The thresholds were set as follows: degree cutoff = 2, node score cutoff = 0.2, k-core = 2, and maximum depth = 100. The gene cluster with the highest score was selected and regarded as Hub genes.

### Relative genes in Hub Genes with FANCI

The positive and negative Hub genes were explored further. KM curves for the OS of these genes in LIHC were constructed, and the genes with p-value < 0.001 were selected. Additionally, a Spearman correlation analysis between FANCI and other Hub genes was performed by ggstatsplot to select the most representative genes, which were also the genes most related to FANCI with high coefficients of correlation. Then, the selected genes were utilized to build a prognostic model using an equation of RiskScore. A time receiver operating characteristic (ROC) analysis (Version: 0.4) was conducted to compare the predictive accuracy and risk score. Using the glmnet (Version: 4.1-1) package of R, the least absolute shrinkage and selection operator (LASSO) regression method was utilized to select the high-risk gene signatures, and the data used was from LIHC RNA-seq data in the TCGA.

### Immune Infiltration

The TIMER2.0 database (http://timer.cistrome.org/) was used to explore immune infiltration among various cancer. The relationship between the expression of FANCI and the infiltration level of immune cells in LIHC was investigated, with B, CD4+ T, Th1, Th2, Treg, NK, M2, DC, and CD8+ T cells being immune cells of interest. Additionally, the infiltration levels of the lymphocytes in FANCI-expressed LIHC patients were explored using the TISIDB website (http://cis.hku.hk/TISIDB/index.php). Then, to demonstrate the impact of FANCI on immune infiltration, a correlation analysis was performed between the expression of FANCI and the expression of immune checkpoints and chemokines. The LIHC mRNA-seq data from the TCGA database was imputed into R software ggstatsplot to conduct Spearman's correlation analysis, which explored the relationship between the expression of FANCI and microsatellite instability in LIHC.

### Western Blotting

The cell line samples were collected and lysed by Radio Immunoprecipitation Assay (RIPA) solution with protease and phosphatase inhibitors to obtain total protein. The BCA protein Assay Kit was utilized to determine the concentration of each sample solution. An equal amount of protein (40μg) for each sample was prepared and denatured. The total proteins were separated by 12.5% Sodium dodecyl sulfate-polyacrylamide gel electrophoresis (SDS-PAGE) and transferred onto the 0.2μm polyvinylidene fluoride (PVDF) membranes. Then, 5% non-fat milk in TBST was used to block membranes for 1 h. Then, the membranes were incubated with primary antibodies overnight at 4°C. After washing with TBST 3 times, an anti-rabbit secondary antibody conjugated with Horseradish peroxidase (HRP) was added and allowed to incubate for 1 h 20 min. The immunoblots were visualized using an imaging system (Bio-red) with UltraSignal Electrochemiluminescence substrate. β-actin was used as a loading control.

### Statistical Analysis

The results of this study were examined by GraphPad Prism 8, the data were expressed as mean ± SEM. The differences analysis between groups was conducted by the Weltch t' test. The prognostic analysis of FANCI was analyzed by the Kaplan-Meier survival curve. The correlations between different proteins were analyzed by Spearman correlation. A p-value of ≤ 0.05 was regarded as statistically significant.

## Results

### The Expression of FANCI in Pan-cancer

A comparison of FANCI expression in the normal tissues and organs to tumorous tissues was investigated to elucidate the significance of FANCI expression on tumor progression. In addition, the RNA-seq data from the HPA was analyzed. Analysis showed the thymus, testis, and bone marrow have high expression levels of FANCI (44.6 nTPM, 32.9 nTPM, 31.7 nTPM, respectively), whereas, in the liver tissue, FANCI expression levels were much lower (2.9 nTPM). Similar trends were observed in hepatocytes and cholangiocytes, with FANCI expression levels of only 0.8 nTPM and 0 nTPM (Supplementary Figure 1A, B). Using multiple websites (GEPIA, UALCAN, TIMER2, XianTao), the diverse mRNA expression patterns of FANCI in multiple cancers were investigated using the data from the TCGA database. Significant levels of FANCI expression were present in almost all cancers. The transcriptional level of FANCI in tumor cells was noticeably higher than those in the corresponding normal tissues, excluding LAML and TGCT.

### Overexpression of FANCI in LIHC

**Figure 1:**
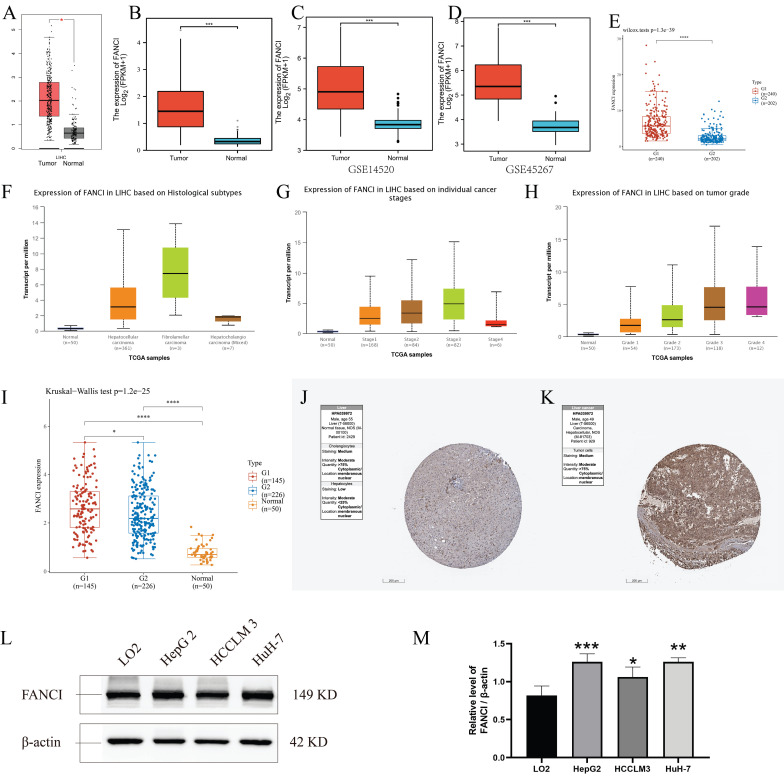
Expression of FANCI in the microarray, immunohistochemical staining datasets, and validations by Western blotting. Scatter diagrams of expression of FANCI in LIHC using RNA-seq data from (A, B) TCGA and (E) ICGC. (C, D) Box plots of expression of FANCI in LIHC using microarray data from GEO datasets (GSE14520, GSE45267). (F, G, H, I) Box plots of expression of FANCI associated with histological subtypes, stage, grade, HBV. (*p < 0.05, ***p < 0.001). (J, K) IHC analysis of LIHC tissue and liver tissue microarray. FANCI expression levels in (L) HCC cell lines. (M) statistical analysis of western blot in L, LO2 cell line was regarded as the control group, Data are expressed as mean ± S.D. Statistical analysis was performed with one-way ANOVA (n = 3; * p < 0.05, ** p < 0.01, *** p < 0.001).

The 529 mRNA-seq values of LIHC patients from the TCGA database were tested using GEPIA. A significant difference in the transcription of FANCI was observed between the samples from LIHC tissue and normal tissue, with FANCI being upregulated in liver cancer (Figure 1A). In addition, two GEO datasets (GSE14520, GSE45267) were studied to validate the expression results found in the TCGA database. As shown in the box plot graphs, FANCI was found to be overexpressed in LIHC patients with a p-value < 0.001. The transcription data from the ICGC databases were also tested. The results parallel those found in the TCGA database. FANCI expression in LIHC is significantly higher than in normal tissue (p= 3e-39) (Figure 1C, D, E).

In addition, the distinct transcription levels of FANCI in diverse LIHC subtypes, stages, and grades were obtained through UALCAN. For the different histological types, results indicated that most liver cancers belonged to hepatocellular carcinoma, which had higher expression of FANCI than normal tissue. Fibrolamellar carcinoma, a rare type of LIHC, expressed more FANCI than HCC (Figure 1F). Furthermore, the highest expression of FANCI was detected in stage 3 LIHC patients. However, increased expression of FANCI in stage 2 LIHC patients indicated a much poorer prognosis than in stage 3. A similar trend was observed in different grades of LIHC. A higher expression of FANCI can be seen in grade 3 patients, and a worse prognosis can be seen in grade 2 patients (Figure 1G, H, Supplementary Figure 1G, H).

In addition, to investigate the impact of the HBV virus on the transcription level of FANCI, mRNA-seq data from the TCGA database was separated into HBV-positive and HBV-negative groups. Plotting into a box chart, the data suggest that the transcription of FANCI was promoted by prior HBV infection in the LIHC patients, and a worse prognostic outcome was observed in OS (log-rank P = 0.00087) (Figure 1I, Supplementary Figure 1I).

Lastly, the protein expression data of FANCI in LIHC patients and normal tissue can be obtained from the HPA, and an increased level of FANCI can be observed in the LIHC tissue compared to normal liver tissue (Figure 1J, K). Furtherly, significantly higher FNACI expression levels can be observed in the LIHC cell lines HepG2 and HuH-7 compared with normal liver cell line LO2 (Figure 1L). besides, in colorectal cancer cell lines, SW620, SW480, HCT116, LS174T cells showed much higher expression of FANCI compared with FHC cells (Supplementary Figure 1J). In Breast invasive carcinoma (BRCA) cell lines, the expression of FANCI in MCF-7 and SK-BR-3 cells exceeded that of normal breast cell line MCF-10A (Supplementary Figure 1K). On the contrary, the normal nasopharyngeal cell line NP69 expressed more FANCI than NPC cell lines CNE1 and CNE2 (Supplementary Figure 1L).

### Prognosis of FANCI in LIHC

**Figure 2:**
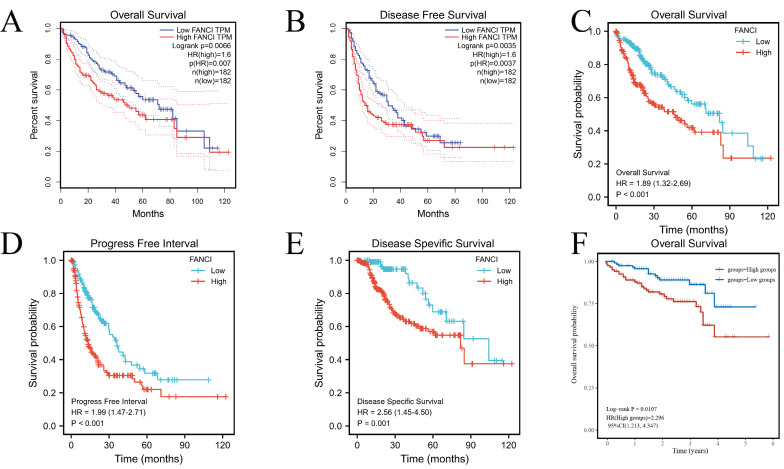
Survival analysis of FANCI in LIHC. (A, C) Overall survival analysis of FANCI in LIHC. (B) Disease Free survival analysis of FANCI in LIHC. (D) Progress-free interval analysis of FANCI in LIHC. (E) Disease Free survival analysis of FANCI in LIHC. (F) Overall survival analysis of FANCI in LIHC using mRNA data from ICGC.

To illuminate the relation between FANCI expression level and the prognosis of LIHC patients, various survival curves were generated to discern the association of FANCI expression levels with the prognosis of LIHC patients. These survival curves were graphed using GEPIA and R package survival and survminer. The TCGA-LIHC data from the GEPIA and R software suggests that high FANCI TPM was correlated with a worse overall survival rate (p=0.007, p<0.001) and a higher risk of death (HR=1.6, HR=1.89) compared with patient expressing low FANCI (Figure 2A, C). The DFS and PFI (p=0.0035, p<0.001) illustrated the periods after treatment in which patients are in total or partial remission without cancer progression. LIHC patients with high FANCI levels (FANCI^high^) suffered faster cancer recurrence than LIHC patients with low FANCI levels (FANCI^low^) (Figure 2B, D). Comparably, the DSS graph (p=0.001) indicated that more LIHC patients with FANCI^high^ died than LIHC patients with FANCI^low^ during the same period (Figure 2E). Ultimately, the OS curves generated from ICGC data indicated a worse prognostic outcome in the LIHC patients with FANCI^high^ compared to those with FANCI^low^ (Figure 2F). In the pan-cancer forest plot, the ACC patients with upregulated FANCI showed an extremely high risk of death (HR = 11.8983). In addition, there was a significant difference in FANCI expression between Brain Lower Grade Glioma (LGG), LIHC, Lung adenocarcinoma (LUAD), and Mesothelioma (MESO), which a p-value < 0.01 (Supplementary Figure 2).

### The associated differential gene related to FANCI in LIHC

**Figure 3:**
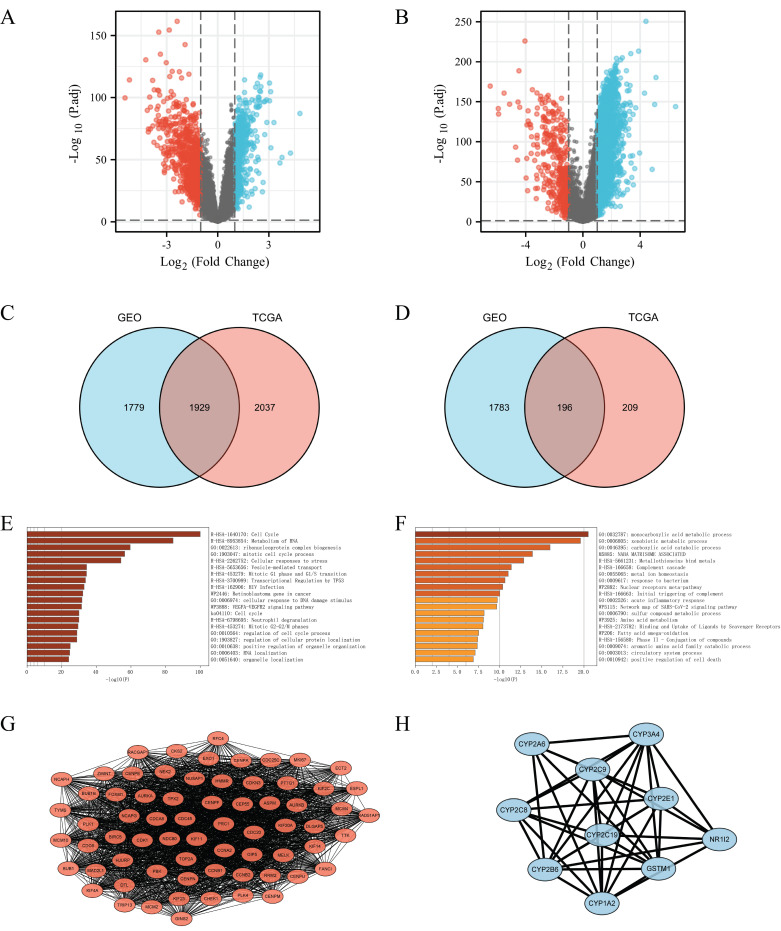
DEGs were selected from the GEO and TCGA datasets with enrichment and PPI network analysis. (A) Volcano plot of differentially expressed genes associated with FANCI in LIHC patients, which were analyzed by using GEO data (GSE14520). Upregulated genes are shown in red and downregulated genes are shown in green. (B) Volcano plot of differentially expressed genes associated with FANCI in LIHC patients, which were analyzed by using TCGA data. Upregulated genes are shown in pink and downregulated genes are shown in blue. (C, D) Overlapping upregulated and downregulated DEGs correlated with FANCI between the TCGA and GEO gene lists. (E, F) Enrichment of function and signaling pathways of positively and negatively correlated DEGs in LIHC. (G) PPI network of the 68 positive Hub genes (MCODE score = 62.388). (H) PPI network of the 10 negative Hub genes (MCODE score = 8.889).

In order to investigate the systemic impact of a group of genes on the development and progression of LIHC, significant differentially expressed genes in the high and low FANCI expression groups were selected. The GEO dataset GSE14520 was processed to obtain the upregulated and downregulated DEGs (adjusted p ≤ 0.05, |log2FC| ≥ 1). A volcano plot was generated with DEGs related to FANCI, with 3,708 genes identified as upregulated, and 1,979 genes identified as downregulated in LIHC (Figure 3A, Supplementary Table 1). Moreover, by using the same cutoff value, TCGA data were applied in the Limma package to get the DEGs correlated with FANCI. From TCGA, there were 3,966 upregulated DEGs and 405 downregulated DEGs in LIHC (Figure 3B, Supplementary Table 2). Subsequently, the DEGs acquired from two databases were compared to find the overlapping DEGs, which would be genes significantly correlated with FANCI. There was an overlap of 1,929 genes for upregulated DEGs, and 196 genes for downregulated DEGs (Figure 3C, D, Supplementary Table 3).

### Functional enrichment analysis of DEGs related to FANCI

To gain insight into the role of FANCI and its positively and negatively correlated genes in the progression and proliferation of LIHC, the 1,929 upregulated and 196 downregulated DEGs were inputted into Metascape to perform KEGG and GO gene enrichment analysis. For the positively correlated genes, as shown in Figure 3 E, FANCI participated in the transition between the G1 to G1/S phase and G2 to G2/M phases, protein localization, and process regulation in the cell cycle. FANCI is also proposed to mediate cellular stress responses, DNA damage repair responses, and transcriptional regulation by TP53. In addition, FANCI also plays a role in the metabolism of RNA, the biogenesis of the ribonucleoprotein complex, and the VEGFA-VEGFR2 pathway. The investigation on the downregulated DEGs indicated certain processes were suppressed due to the expression of FANCI. Such processes include the metabolism of carboxylic acid, amino acid, and fatty acid. Moreover, the activation of the complement cascade and the acute inflammatory response were also reduced (Figure 3F).

The PPI networks were constructed using String and visualized by Cytoscape (Supplementary Figure 3). For the upregulated PPI network, 1,928 nodes, and 17,913 edges were acquired with the thresholds. MCODE utilized the networks to identify the most notable cluster, which contained 68 nodes and 2,090 edges with a score of 62.388 (Figure 3G). Similarly, the downregulated network of 196 nodes and 193 edges was analyzed, and the output was a cluster with an MCODE score of 8.889 with only ten genes (Figure 3H). These genes would be regarded as positive and negative Hub genes for downstream analysis.

### Key genes analysis

**Figure 4:**
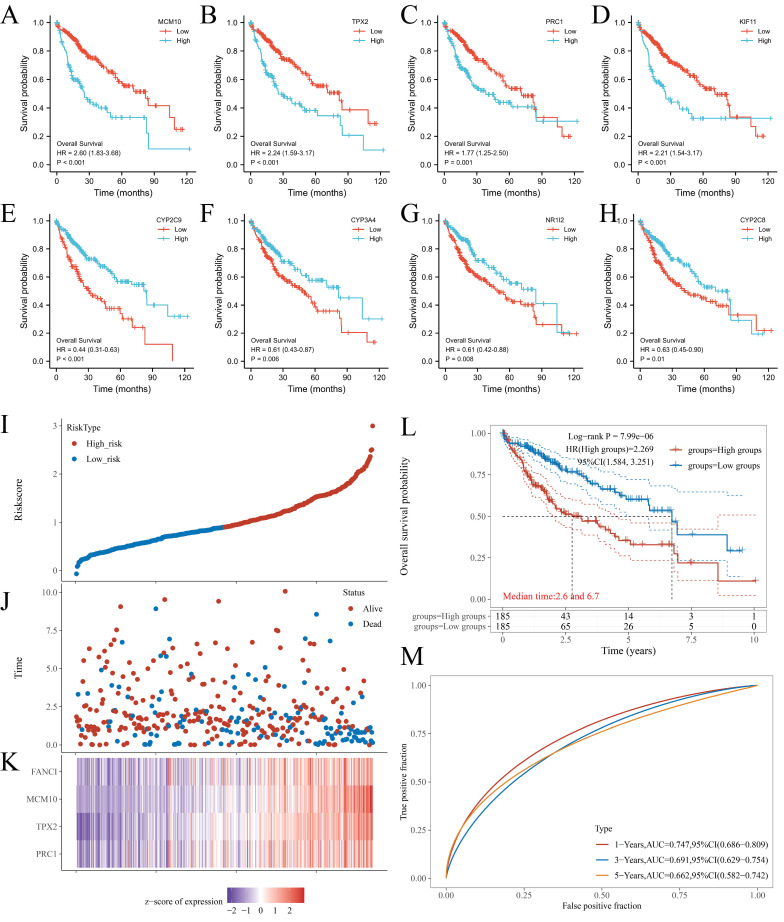
KM plots of OS of key genes in LIHC patients. (A) MCM10; (B) TPX2; (C) PRC1; (D) KIF11; (E) CYP2C9; (F) CYP3A4; (G) NR1I2; and (H) CYP2C8. (I) Dotted curve of risk score. (J) Scatter diagram of survival status. (K) Heatmap of the gene expression pattern. (L) Overall survival analysis of the prognostic model. (M) Time-dependent ROC analysis, 1-, 3-, 5-years.

From the PPI network, 68 positive Hub genes and 10 negative Hub genes were acquired. To select genes that significantly impacted the prognostic outcome, these Hub genes were evaluated by performing univariate Cox regression and plotting KM curves. The result was a selection of genes that acted as representatives with a p-value of ≤ 0.001 for the selected positive Hub genes (32 genes) and ≤ 0.05 for the selected negative Hub genes (5 genes). Subsequently, the correlation between the expression of the selected Hub genes and FANCI was evaluated, resulting in the identification of four key genes that had a close link with FANCI. For the positive Hub genes, MCM10 (R = 0.92), TPX2(R = 0.91), PRC1(R = 0.94) and KIF11(R = 0.91) have a strong correlation with FANCI and lead to poor prognosis in LIHC, as shown in Figure 4 A-D. As for negative Hub genes, CYP2C9, CYP3A4, NR1I2, and CYP2C8 were selected. Low expression of these genes in LIHC would lead to poor prognosis (Figure 4E-H).

The four key upregulated genes associated with FANCI were used to establish a multivariate prognostic model using TCGA data. The risk score can be represented by the equation: (-0.3972) *PRC1 + (0.4552) *TPX2 + (0.4643) *MCM10 + (-0.1587) *FANCI. Furthermore, based on the medium risk score, samples would be divided into high-risk and low-risk. A dotted risk score curve, a scatter diagram of survival status, and a heatmap of the gene expression pattern were generated (Figure 4I-K). The OS line illustrated that a higher expression of key genes was associated with a higher risk of death and, consequently, a worse prognosis for LIHC (Log-rank P = 7.99e-06) (Figure 4L). Lastly, the Area Under Curves (AUCs) of ROC curves for 1-, 3-, and 5-years overall survival were calculated as 0.747, 0.691, and 0.662, respectively, which suggest that this prognostic model can reliably predict the risk of patients (Figure 4 M).

### The immune infiltration status in LIHC associated with FANCI high expression

**Figure 5:**
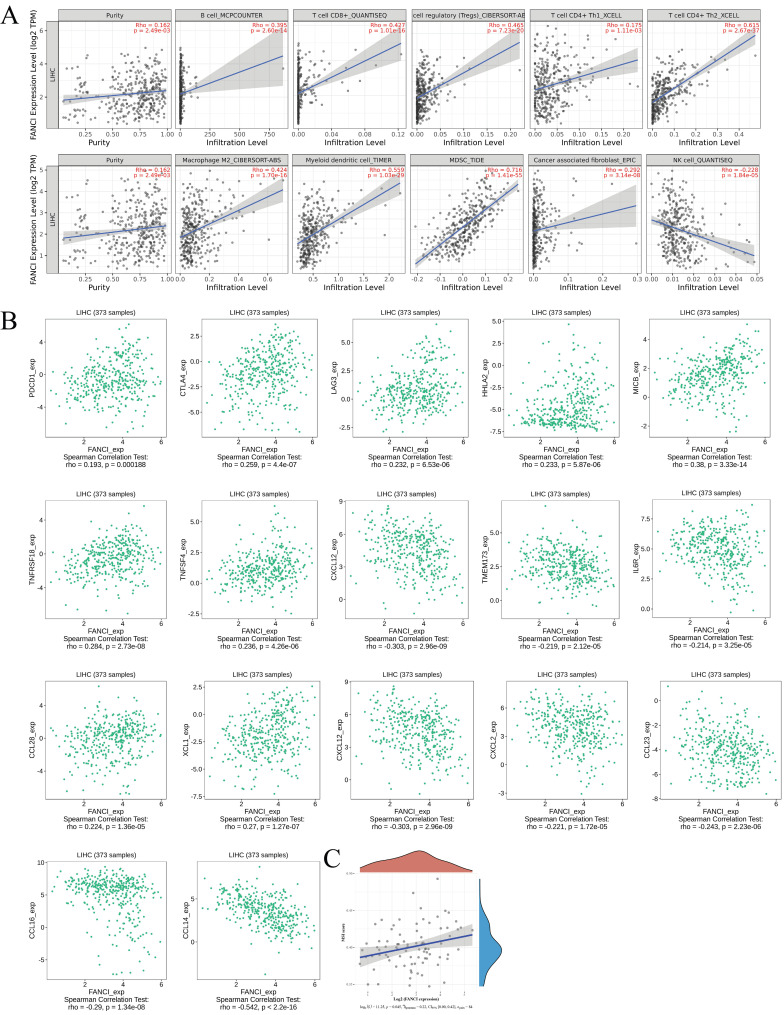
Immune infiltration state associated with the FANCI in LIHC. (A) Scatter plots of the level of tumor-infiltrating immune cells correlated to the expression of FANCI in LIHC. (B) Scatter plots of the expression of immune molecular correlated to the expression of FANCI in LIHC. (C) Scatter plots of the relationship between the expression of FANCI and MSI level in patients with LIHC.

The tumor microenvironment plays a vital role in tumor progression, metastasis, and immunotherapy efficacy. Thus, to investigate the relation between the high FANCI expression and the immune infiltration level in LIHC, COX regression was used to assess the correlation between FANCI expression and lymphocytes, immune checkpoints, and chemokines via TIMER2 and TISIDB. The results suggested that high FANCI expression was positively correlated with the number of B, cytotoxic CD8+ T, Tregs, Th2, macrophage M2, DC, and myeloid-derived suppressor cells (MDSCs), in contrast, there was a negative correlation between high FANCI expression and the number of Natural Killer cells (NK cells) (Figure 5A). The expression of FANCI was also assessed for its correlation with immune checkpoints, which are essential for immunosuppression and immune evasion. The inhibitory immune checkpoints PDCD1, CTLA4, and LAG3 showed positive correlations with the expression of FANCI. For the immunostimulatory checkpoints, positive correlations were observed in HHLA2, MICB, TNFRSF18, and TNFSF4, and negative correlations were detected in TMEM173, IL6R, and CXCL12. As for chemokines, which participate in the recruitment and trafficking of lymphocytes, FANCI expression upregulated CCL28 and XCL1 and suppressed CCL14, CCL16, CCL23, CXCL2, and CXCL12 (Figure 5B). Lastly, the microsatellite instability (MSI) status was evaluated, with the scatter plot proposing that the ascending MSI score was positively correlated with the increased expression of FANCI (Figure 5C).

## Discussion

From 2013 to 2017, the incidence rates of cancer in both sexes remained stable, while the cancer death rate consistently declined by around 1.9% per year during the five-year span from 2014 to 2018^14^. Thanks to the progress made in diagnostic and treatment technologies, tumors have become increasingly treatable. Nonetheless, the incidence and death rates of liver cancer are still rising. This hints that liver cancer is a more complicated and aggressive type of cancer associated with poor survival rates and prognosis. The treatment strategies for liver cancer can be either selective or non-selective. Classical treatments such as surgery, chemotherapy, and radiotherapy are applied to all kinds of liver cancer patients without subtyping, leading to unwanted systemic side effects and a high risk of recurrence 15. Sorafenib, a first-line chemotherapeutic, has been found to be effective in less than 33% of patients, and chemoresistance rapidly appeared within six months 16. Similarly, the objective response rate for targeted therapy or immunotherapy remains inadequate, which can be attributed in part to the broad nature of current subtypes used for treatment selection. To address this issue, more specific and distinct subtypes can be utilized to better match patients with targeted treatments or personalized therapies that are more likely to be effective. The selection of treatment plans for each patient relies on specific biomarkers, which can provide insight into the presence, progression, prognosis, and treatment of cancer. To explore key biomarkers, we conducted a bioinformatics analysis to evaluate the various roles of FANCI in LIHC.

FANCI plays an essential role in identifying and further recruiting repair proteins to facilitate their repair. As for the other half of the FANCD2-FANCI dimer, FANCD2 was reported as upregulated in melanomas and colorectal carcinoma and was associated with aberrant cell cycle and proliferation 17. This suggests that the FA family of proteins and the correlated DNA damage response (DDR) contribute significantly to cancer cell survival and prognosis. In melanoma, the knockdown of FANCD2 suppressed the migration and proliferation of the tumor cells 18. Seeing as FANCI has a similar function to FANCD2 in the DDR pathway and is closely related, it is necessary to investigate the role of FANCI in cancer. Therefore, we utilized bioinformatic tools to explore the potency of FANCI as a biomarker for prognosis or therapeutic targets.

Analysis of clinicopathological information has revealed that the expression of FANCI is associated with factors such as the age, stage, and grade of the patient. 68.5% of G3 patients and 64.7% of stage III patients exhibit high levels of FANCI expression. With the progression of LIHC, the expression of FANCI is upregulated, which may indicate that FANCI contributes to cancer progression. A study suggested that FANCI promotes the proliferation of the LUAD cells by activating MEK/ERK and MMPs pathway 19. Meanwhile, the poor differentiation is accompanied by the high expression of FANCI, which in turn promotes migration and metastasis, resulting in a poor prognosis. In addition, FANCI was found to be highly expressed in HBV-positive LIHC, which is associated with poor overall survival. The relationship between HBV infection and LIHC had been investigated. As the genetic materials of the HBV integrate into the host genome, it can mutate cancer-related genes such as proto-oncogenes and can induce tumorigenesis and subsequent proliferation of the cancer cells 20. At the same time, the activation of the cell cycle pathway is commonly found in HBV-positive LIHC 21. Thus, HBV infection may stimulate the expression of FANCI to facilitate the integration of the HBV genome and repair the DNA damage during insertion.

According to the results of gene enrichment analysis, the upregulated DEGs mediated the regulation of the cell cycle, biogenesis of ribonucleoprotein (RNP) complex, VEGFR pathway, and transcriptional regulation of TP53. RNPs have been found to participate in multiple facets of cancer progression. Some RNPs such as RBM3, IMP3, and CUG-BP1 facilitate the formation of drug resistance 22 while heterogeneous nuclear ribonucleoprotein K has been associated with cancer procession and high risk of metastasis 23. Telomerase is also an RNP, which allows for tumor cell immortalization 24. The activation of VEGFR triggers angiogenesis, which facilitates the growth and acquisition of nutrients in tumor cells. Dysfunctional VEGFR has been found in various types of cancer 25. In LIHC, sorafenib could be utilized to inhibit the activation of multiple kinases, including VEGFR, MEPK, and PDGFR 26. The upregulation of the VEGFR pathway is related to the expression of FANCI, which allows for combination therapy using an anti-VEGFR agent and an anti-FANCI agent. Additionally, retinoblastoma gene expression was enhanced, and RB1 was found in the upregulated gene list. The RB protein can suppress growth-promoting proteins to trigger cell division. Thus, overexpression of the RB gene would lead to uncontrolled cell proliferation 27. The transition of different phases of the cell cycle is controlled by the cyclin-dependent kinases (CDKs) and the polo-like kinases (PLKs). CDK1, CDK4, CCNE1, CDKN2A, and PLK1 were all detected in the overlapping positively correlated gene list. CDK1 was reported to play an essential role in the formation and growth of cancer 28. Amplification of CDK4 and CCNE1 is frequently observed in cancer, promoting the development and progression of cancer 29, 30. CHK1 can be regarded as an initiator of the FA pathway and was increasingly expressed in LIHC, which promotes cancer survival and progression via enabling DNA damage repair. Subsequently, PLK1 can unlock the G2 phase arrest caused by DNA damage. Moreover, it can override the cell cycle checkpoints, which can lead to genetic instability 31.

The overexpression of FANCI, CHK1, and PLK1, as well as the overactivation of the DDR pathway, cooperate to promote the cell cycle process and the division of the tumor cells. They also facilitate tumor cells to resist and repair the damage caused by therapeutic agents. A study regarded ICLs as the most damaging and lethal form of DNA damage to cells 32. In this study, researchers explored and developed four kinds of crosslinking agents, namely nitrogen mustards, mitomycin, psoralens, and platinum-based compounds. Cisplatin was mainly studied, with a powerful capacity to bend and unwind DNA strands 7. However, a high rate of chemoresistance to cisplatin has been observed in LIHC 33. Chemoresistance could be mediated by the DDR involving FANCI or other DDR proteins. Therefore, blocking those proteins may reverse the acquired chemoresistance in cancer cells. PARP inhibitors, nucleotide excision repair inhibitors, DDR kinase, and pathway inhibitors were investigated with exciting outcomes 34. Similarly, anti-FANCI inhibitors have the potential to overcome chemoresistance in LIHC, and combined with crosslinking agents have the potential to kill chemoresistant cancer cells. Additionally, inhibition of FANCI could sensitize cancer cells to crosslinking agents, which would reduce the dosage and side effects of crosslinking agents. Thus, the high expression of FANCI hints at LIHC that are resistant to ICL agents but may be sensitive to other DNA damage agents. It can also be a biomarker to select patients who can benefit from crosslinking agents.

From the GO analysis, immune system processes were upregulated (Supplementary Figure 4 A). Thus, to examine the link between FANCI expression and immune infiltration level, we investigated the levels of immune cells and correlated molecules in LIHC. Typically, the immune system can monitor pathogens and abnormal cells, which have the risk of carcinogenesis. However, various immune cells have a double-edged effect regarding cancer cells. CD8+ T cells and NK cells prevent tumor cells through multiple mechanisms that induce cell death. MDSCs, Tregs, M2, and Th1/2 cells can promote tumor progression and also facilitate immune evasion. It can be observed that the levels of B cells and CD8+ T cells were ascending in the FANCI highly expressed LIHC patients. They constitute the majority of tumor-infiltrating immune cells (TILs) and work to eliminate tumor cells. CD8+ T cells recognize tumor antigens presented by MHC I molecular and thus activate to kill tumor cells via cytotoxic granules or FasL-mediated apoptosis 35. Similarly, activated B cells can produce antibodies against tumor cells, strengthen CTLs activity, or excrete granzyme B to kill tumor cells directly 36. However, high levels of the tumor-infiltrating B cells, especially regulatory B cells (Bregs), drive tumor progression by producing immunosuppressive cytokines, such as IL10 and TGF-β, which are found to be simultaneously expressed with FANCI (Supplementary Figure 4 B, C). Some studies indicated that the presence of TIBs directly suppressed the function of the CTLs 37. Additionally, Tfh cells also were increased in LIHC, which are necessary for activating TIBs and have immunosuppression capabilities 38. M2 and Treg cells would be induced by TGF-β and IL-10 from Breg cells. M2 cells, in turn, produce anti-inflammatory cytokines and chemokines to promote epithelial-mesenchymal transition, facilitate tumor invasion and metastasis, and suppress the activities of CTLs 39. Furthermore, Treg cells utilize CTLA4 to directly inhibit the function of the CTLs, which was also found to be positively correlated with FANCI. Th2 cells play a pro-tumorigenic role by secreting IL-4, IL-5, and IL-13, which promote tumor growth and metastasis. Additionally, elevated IL-4 suppresses the differentiation of Th1 cells, which facilitates the differentiation and activation of CD8+ T cells. IL-4 also triggers the STAT6 signaling pathway, which enhances the function of M2 cells to assist in the growth of tumors 40, 41. MDSCs produce IL-6 to promote EMT and participate in the formation of pre-metastatic niche. Moreover, they can activate Tregs and Th2 cells to inhibit CTLs and NK cells respectively 42, 43. Seemingly contradictory, FANCI expression is positively linked to the level of CD8+ T cells. However, the prognosis in LIHC was still poor. As mentioned above, overexpression of FANCI increased the levels of several immunosuppressive cells. FANCI could induce some downstream pathways to stimulate the infiltration and activity of immunosuppressive cells. These immunosuppressive cells then coordinately restrict the proliferation and activation of the CD8+ T cells and infiltration of the NK cells. This could contribute to the poor prognosis observed in LIHC despite the positive association between FANCI expression and CD8+ T cell levels.

Furthermore, immune checkpoints, essential molecules in the signaling pathway, are vital in maintaining immune homeostasis and regulating immune response through both promotion and inhibition. Conversely, abnormal expression of these checkpoints in cancer cells or immune cells inhibits antitumor immunity, leading to immune evasion and rapid tumor growth. PD-1, CTLA-4, and LAG3 were upregulated in FANCI^high^ LIHC, and they exert inhibitory function to CTLs relating to proliferation, survival, and production of cytokines. Therefore, to reverse this suppression of tumor immunity, antibody-based immune checkpoint inhibitors were designed and developed. Pembrolizumab and Nivolumab, anti-PD-1 ICIs, were approved to treat HCC 4, 44. Ipilimumab, an anti-CTLA-4 ICI, was authorized for combination therapy with Nivolumab for HCC patients 45. Due to the high-level infiltration of CD8+ T cells and overexpression of the inhibitory immune checkpoints with an increasing level of MSI in FANCI^high^ LIHC patients, it offers an excellent opportunity for the application of immunotherapies such as ICIs and chimeric antigen receptor T (CAR-T) therapy. As FANCI is mainly expressed in the cell nucleus, classical CAR-T will not work as it interacts with membrane proteins. Yarmarkovich, Mark, et al. utilized tumor antigen peptides on MHC-1 to construct peptide-centric CAR-Ts that can recognize oncoproteins present inside tumor cells 46.

High expression of FANCI in LIHC is associated with a poor prognosis and contributes to tumor proliferation and metastasis. Its role in the DDR pathway promotes chemoresistance. Additionally, FANCI also participates in immunosuppression and is correlated with elevated levels of TILs. Therefore, anti-FANCI inhibitors could be developed, which not only directly suppress cell viability and tumor metastasis, but also reactivates susceptibility to chemotherapy and immunotherapy in LIHC cells. A combination of different agents could possess higher efficiency in eliminating tumor cells. Lastly, the mechanism by which FANCI regulates proliferation and immunosuppression requires further extensive research.

## Conclusions

In conclusion, we investigated the capabilities of FANCI as a prognostic and therapeutical biomarker in live hepatocellular carcinoma. FANCI participates in the FA pathway, which is responsible for DNA damage repair. Thus, FANCI is a crucial gene facilitating tumor proliferation and metastasis. Furthermore, FANCI plays a role in the emergence of chemoresistance and immunosuppression, which leads to poor prognosis in LIHC. Future research should focus on exploring the efficacy of anti-FANCI inhibitors and developing optimal multimodal therapy.

## Supplementary Material

Supplementary figures.

Supplementary tables.

## Abbreviations

LIHC: Liver Hepatocellular Carcinoma
DEGs: differentially expressed genes
PPI: Protein-Protein interaction
MCODE: molecular complex detection
HBV: hepatitis B virus
Tregs: regulatory T cells
Th2: CD4+ T helper 2 cells
HCC: hepatocellular carcinoma
FDA: Food and Drug Administration
ICI: immune checkpoint inhibitor
FA: Fanconi anemia
ICLs: ,interstrand cross-links
ATR: Rad3-related kinase
CHK1: checkpoint kinase 1
OS: overall survival
DFS: disease-free survival
HR: hazards ratio
HPA: Human Protein Atlas
CI: Confidence interval
DSS: disease specific survival
KEGG: Kyoto Encyclopedia of Genes and Genomes
GO: Gene Ontology
ROC: receiver operating characteristic
LASSO: least absolute shrinkage and selection operator regression
SDS-PAGE: Sodium dodecyl sulfate polyacrylamide gel electrophoresis
PVDF: polyvinylidene fluoride
HRP: Horseradish peroxidase
BRCA: Breast invasive carcinoma
LGG: Lower Grade Glioma
LUAD: Lung adenocarcinoma
MESO: Mesothelioma
AUCs: Area Under Curves
NK cells: Natural Killer cells
MDSCs: myeloid-derived suppressor cells
MSI: microsatellite instability
DDR: DNA damage response
RNP: ribonucleoprotein
PLKs: polo-like kinases
CDKs: cyclin-dependent kinases
TILs: tumor-infiltrating immune cells
Bregs: regulatory B cells
CAR-T: chimeric antigen receptor T
