# Supplementary material for: FANCI is Associated with Poor Prognosis and Immune Infiltration in Liver Hepatocellular Carcinoma
Source: Int J Med Sci. 2023 May 12;20(7):918–32. doi: 10.7150/ijms.83760 (PMC10266051; doi:10.7150/ijms.83760)
Supplement: Supplementary file 1 — Supplementary figures. [file ijmsv20p0918s1.pdf]

Supplementary Material

FANCI is associated with poor prognosis and immune infiltration in liver hepatocellular carcinoma

Yibo Hou[1], Jianing Li[2], Albert Yu[1], Kexin Deng[1], Jiawei Chen[1], Zixian Wang[1], Laiqiang Huang[1*], Shaohua Ma[1*], Xiaoyong Dai[1*]

*** Correspondence:** Laiqiang Huang, huanglq@tsinghua.edu.cn; Shaohua Ma, ma.shaohua@sz.tsinghua.edu.cn; Xiaoyong Dai, dai-xy18@tsinghua.org.cn.

1.1 Supplementary Figures

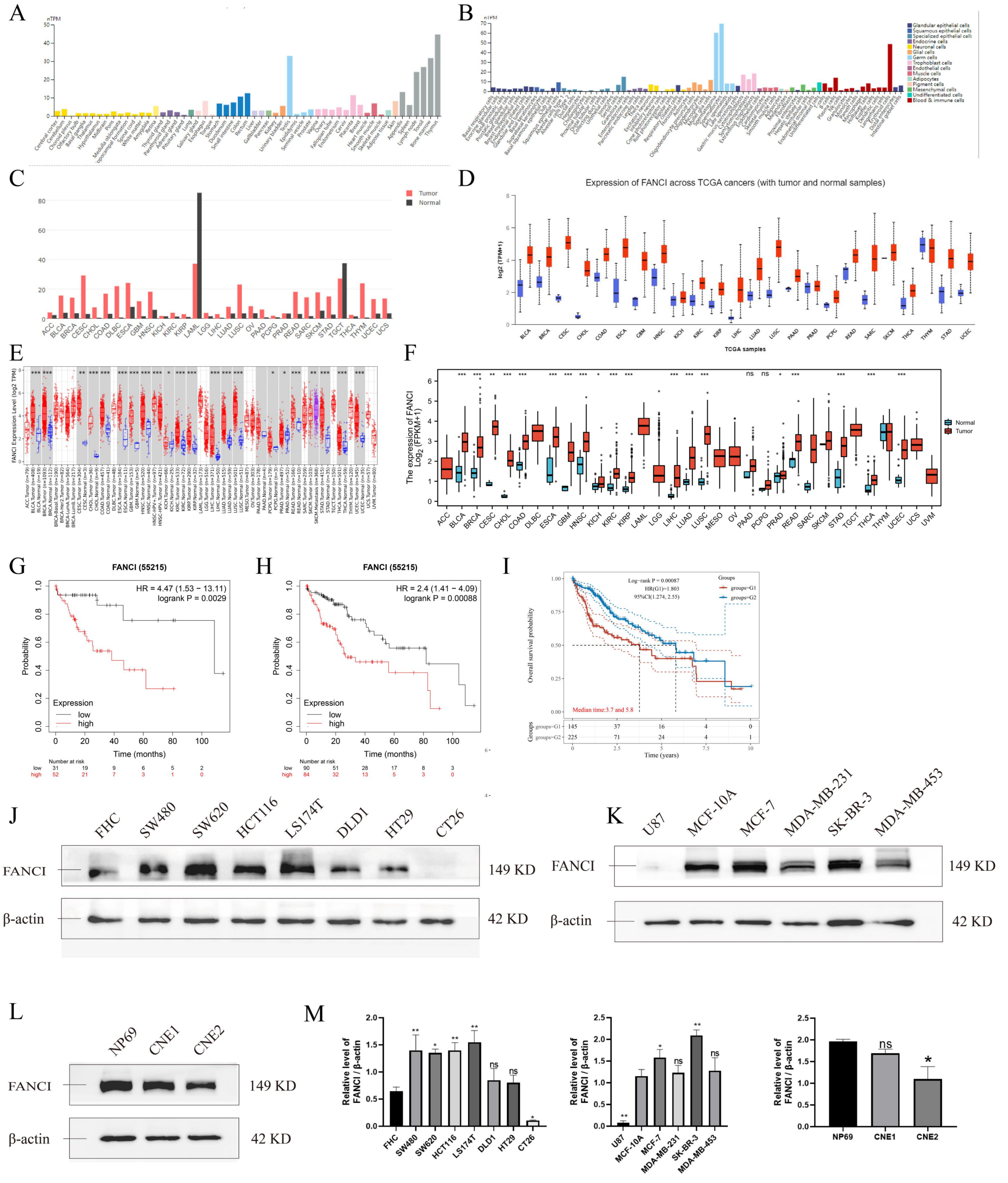

Supplementary Figure 1. (A, B) Bar charts of expression of FANCI in variable tissue and variable cells. (C-F) Box plots of expression of FANCI in the Pan-cancer. (G, H, I) Overall survival curves of FANCI in stage II, grade II and HBV patients. (*p < 0.05, ***p < 0.001). (M, N) IHC analysis of LIHC tissue and liver tissue microarray. FANCI expression levels in (J) CRC cell lines, (K) BRCA cell lines and GBM cell line U87, (L) NPC cell lines, (M) statistical analysis of western blot in J, K, L, FHC, MCF-10A, NP69 cell lines were regarded as control group Data are expressed as mean ± S.D. Statistical analysis was performed with one-way ANOVA (n = 3; * p < 0.05, ** p < 0.01, *** p < 0.001).

Cancer	Pvalue	Hazard Ratio(95% CI)
ACC	<0.0001	11.8983(4.05315,34.92831)
BLCA	0.8771	1.02356(0.76199,1.37491)
BRCA	0.1209	1.28567(0.93592,1.76611)
CESC	0.5732	0.87525(0.55055,1.39144)
CHOL	0.3346	1.63701(0.60152,4.45503)
COAD	0.8904	1.0277(0.69673,1.51587)
DLBC	0.4923	0.60405(0.14329,2.54635)
ESCA	0.4614	1.2034(0.73522,1.96971)
GBM	0.4981	0.88193(0.61313,1.26856)
HNSC	0.7746	1.03973(0.7963,1.35759)
KIRC	0.1827	1.22563(0.90866,1.65317)
KIRP	0.0684	1.75892(0.95835,3.22827)
LAML	0.6712	0.91283(0.59907,1.39092)
LGG	0.0011	1.85967(1.28076,2.70026)
LIHC	0.0015	1.76108(1.24131,2.49848)
LUAD	0.0058	1.51014(1.12673,2.02403)
LUSC	0.2554	0.85484(0.6524,1.1201)
MESO	1e−04	2.84658(1.69735,4.77391)
OV	0.1263	0.81633(0.62933,1.05889)
PAAD	0.0371	1.55925(1.02693,2.3675)
PRAD	0.9332	1.05478(0.30323,3.66904)
READ	0.0707	0.47535(0.21219,1.0649)
SARC	0.0142	1.65373(1.10621,2.47223)
SKCM	0.0126	1.41524(1.07716,1.85943)
STAD	0.0823	0.74679(0.53725,1.03804)
TGCT	0.7827	1.3271(0.17769,9.91166)
THCA	0.5491	0.73924(0.27515,1.98614)
THYM	0.0371	0.18141(0.03644,0.90309)
UCEC	0.3206	1.23307(0.81556,1.86432)
UCS	0.8281	0.92782(0.47182,1.82452)
UVM	0.0562	2.38724(0.97735,5.831)

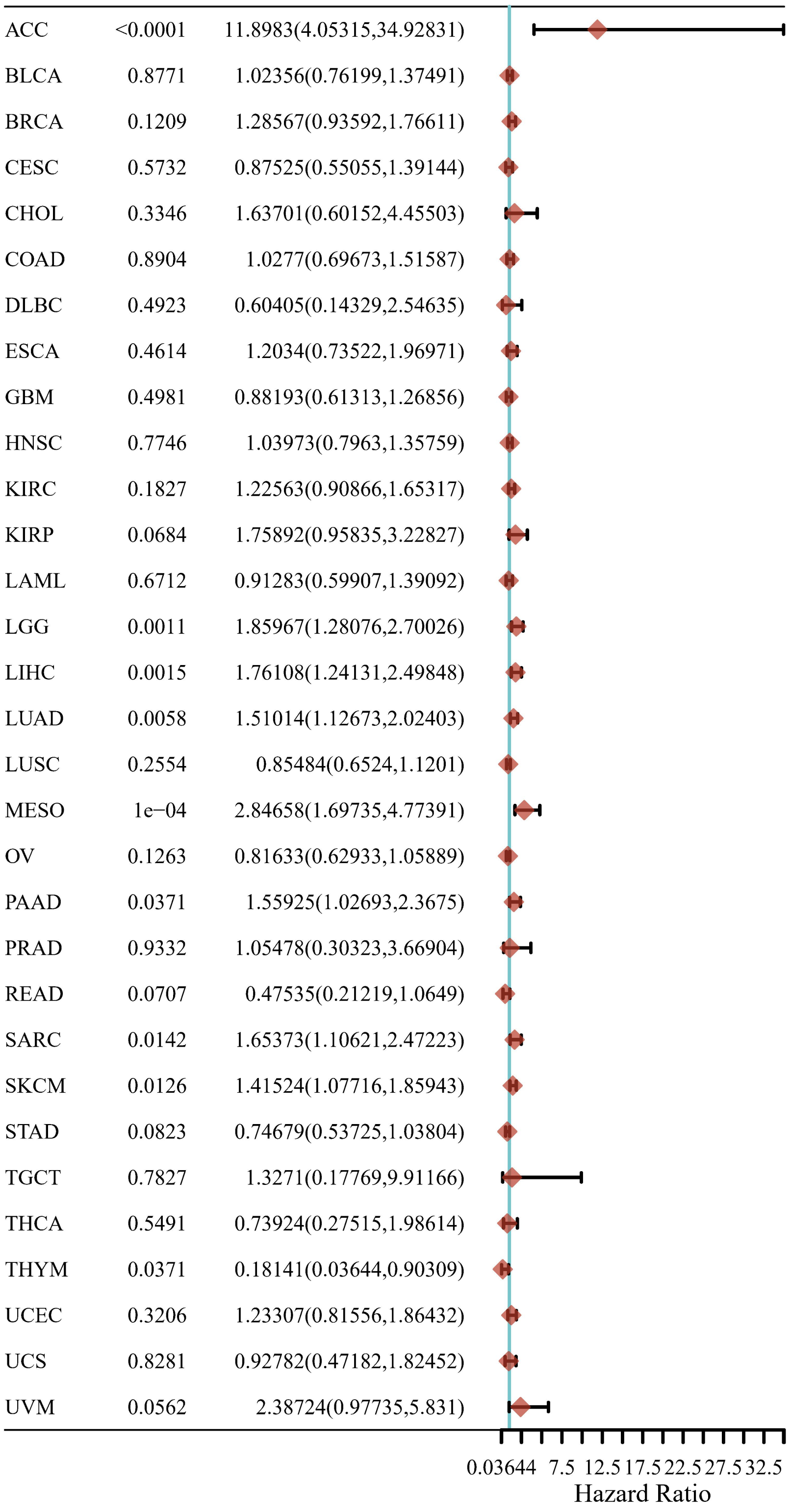

Supplementary Figure 2. Forest plot of prognostic characteristics of FANCI in 31 kinds of cancers.

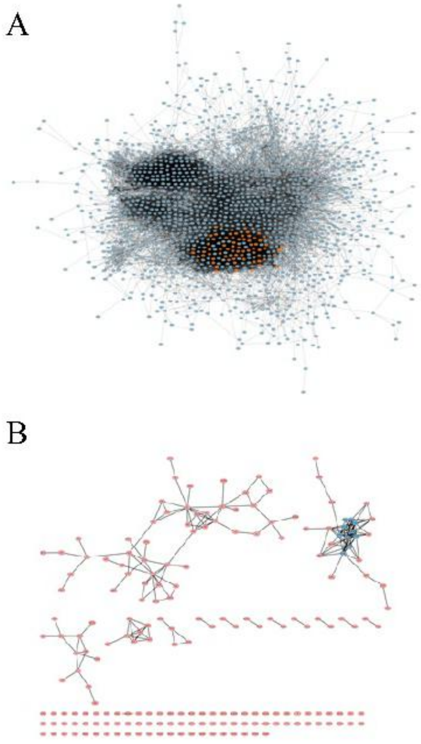

Supplementary Figure 3. PPI network of upregulated and downregulated genes (A, B).

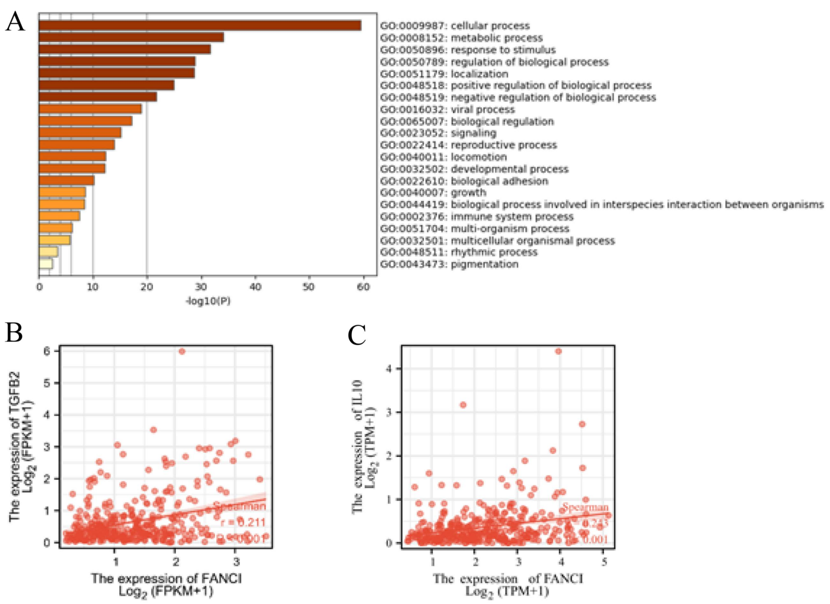

Supplementary Figure 4. (A) the GO analysis of positively correlated DEGs in LIHC. (B) the relationship between FANCI and TGF-β. (C) the relationship between FANCI and IL-10.
